# Supplementary material for: Body Fat Is Superior to Body Mass Index in Predicting Cardiometabolic Risk Factors in Adolescents
Source: Int J Environ Res Public Health. 2023 Jan 23;20(3):2074. doi: 10.3390/ijerph20032074 (PMC9915438; doi:10.3390/ijerph20032074)
Supplement: Supplementary file 1 [file ijerph-20-02074-s001.zip › ijerph-2101052-supplementary.pdf]

Table S1. Association of cardiometabolic risk factors with the BMI and %GC. Viçosa-MG, Brazil, 2010 to 2015 (n = 1043).

	**BMI ***		**%GC ****	
**Risk Factors**	**Odds Ratio (IC95%)**	**Value of p**	**Odds Ratio (IC95%)**	**Value of p**
**SBP**	1.06 (1.04–1.08)	<0.001	-	-
**HDL**	0.97 (0.95–0.98)	<0.001	-	-
**HOMA-IR**	2.19 (1.82–2.64)	<0.001	1.85 (1.55–2.22)	<0.001
**DBP**	-	-	1.06 (1.04–1.08)	<0.001

*BMI <1 considered as a reference. ** %GC < 25% for girls and < 20% for boys considered as reference.
Legend: BMI: Body Mass Index; HDL: High Density Lipoprotein; HOMA-IR: Homeostasis Model Assessment – Insulin Resistance; SBP: Systolic Blood Pressure; DBP: Diastolic Blood Pressure.
